# Pulmonary Arteriovenous Malformation Misdiagnosed As Chronic Obstructive Pulmonary Disease: A Case Report

**DOI:** 10.7759/cureus.111681

**Published:** 2026-06-28

**Authors:** Sujeirys Paulino, Diaz Saez Yordanka, Dmitry Lvovsky

**Affiliations:** 1 Internal Medicine, BronxCare Health System, New York, USA; 2 Pulmonary Medicine, Bronx Care Health System, New York, USA; 3 Pulmonary Medicine, BronxCare Health System, New York, USA

**Keywords:** arterio venous malformations, interventional radiology guided embolization, pulmonary arteriovenous malformation, refractory hypoxemia, vascular anomaly

## Abstract

Pulmonary arteriovenous malformations (PAVMs) are rare vascular anomalies characterized by direct communication between the pulmonary arteries and veins, resulting in a right-to-left shunt and impaired gas exchange. Most cases are associated with hereditary hemorrhagic telangiectasia (HHT), while sporadic, complex lesions are uncommon and can be diagnostically challenging when clinical features overlap with more prevalent pulmonary disease.

This is the case of a chronic smoker who was referred for pulmonary evaluation for suspected chronic obstructive pulmonary disease (COPD). She was found to have hypoxemia with cyanosis and digital clubbing. CT angiography (CTA) of the chest demonstrated right middle and right lower lobe PAVMs. Two consecutive interventions were performed to treat the malformations. She had persistent post-procedure hypoxemia, and a third intervention identified an additional large inferolateral right middle lobe PAVM with a smaller contributing branch, making this a case of a complex PAVM, which is an uncommon occurrence in patients without HHT.

## Introduction

Pulmonary arteriovenous malformations (PAVMs) are rare abnormalities in which there is a direct connection between veins and arteries, providing a communication between the pulmonary and systemic circulations, bypassing the pulmonary capillaries and producing a right-to-left shunt that impairs gas exchange. PAVMs can occur in approximately one in 2,600 individuals in the general population and may be congenital or acquired. Congenital PAVMs are most commonly associated with hereditary hemorrhagic telangiectasia (HHT); meanwhile, acquired causes are associated with traumas, malignancies, liver cirrhosis, mitral stenosis, and infections; sporadic cases are rare [1-3]. Anatomically, when a single segmental artery supplies the malformation, it is referred to as a simple PAVM, accounting for around 89% of cases; when two or more segmental arteries supply the malformation is called a complex PAVM [4].

We present the case of a patient who was initially referred to our clinic for evaluation of suspected chronic obstructive pulmonary disease (COPD). She complained of worsening dyspnea for around six months, intermittent cough productive of whitish sputum, and declining exercise tolerance. Further investigation revealed the presence of sporadic, complex PAVM in the absence of identifiable hereditary or acquired predisposing factors.

## Case presentation

The patient is a 71-year-old woman, a chronic smoker with eleven pack-years, with a medical history of hypertension, dyslipidemia, vitamin D insufficiency, chronic kidney disease, and suspected COPD, who was referred to the pulmonary clinic for evaluation. The patient presented with complaints of worsening shortness of breath for six months, decreased exercise tolerance, being able to walk less than a block, and a chronic intermittent cough with whitish sputum.

Physical examination revealed peripheral cyanosis and digital clubbing. Vital signs were significant for a peripheral oxygen saturation (SpO_2_) of 75% in room air, which partially improved with oxygen supplementation. Due to severe hypoxia, the patient was referred to the hospital for admission. Initial X-ray of the chest reported the presence of mild diffuse bilateral interstitial prominence, with mild superimposed right basilar opacification suggestive of infiltrate/atelectasis (Figure 1).

**Figure 1:**
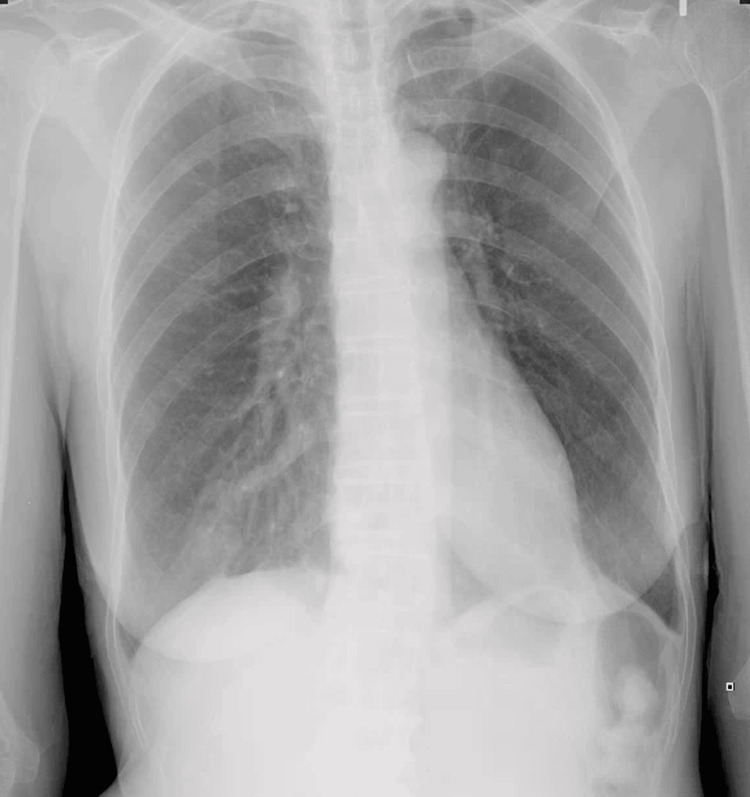
CXR on admission CXR demonstrating mild diffuse bilateral interstitial prominence with subtle right basilar opacities, suggestive of infiltrate or atelectatic change. These findings were subsequently better characterized on CTA as a right lower lobe PAVM. CXR: Chest X-ray; CTA: CT angiography; PAVM: Pulmonary arteriovenous malformation

A CT angiography (CTA) of the chest revealed right middle and right lower PAVMs (Figure 2).

**Figure 2:**
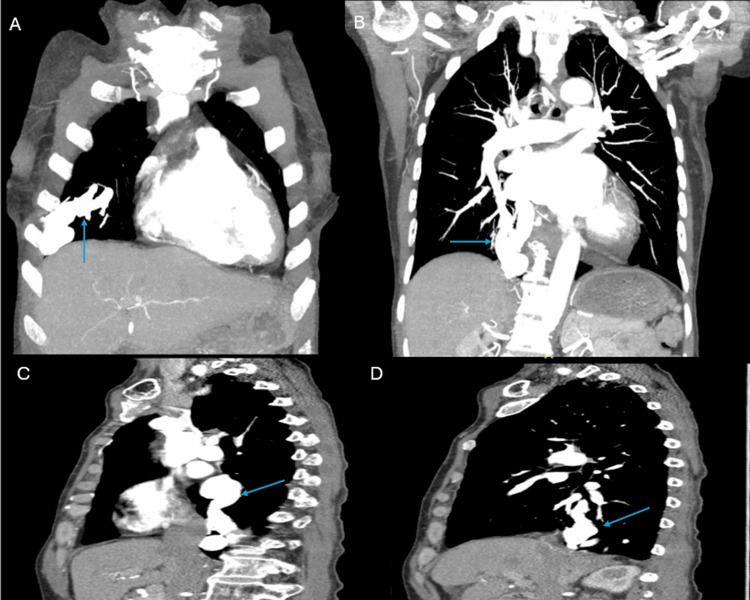
Initial CTA CTA of the chest demonstrating large, right middle, and right lower lobe PAVMs (blue arrows). Panels A, B show coronal views, and panels C, D show lateral views. CTA: CT angiography; PAVM: Pulmonary arteriovenous malformation

Interventional radiology successfully embolized the PAVM located at the interlobar branch of the middle lobe pulmonary artery. However, despite multiple attempts, the one in the right lower lobe could not be embolized due to its high flow and size, prompting balloon-assisted embolization (Figure 3).

**Figure 3:**
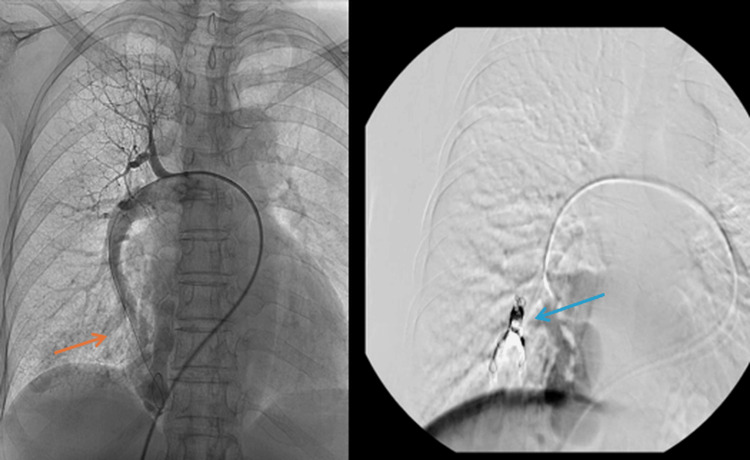
Flouroscopy Fluoroscopy demonstrating a PAVM. Left panel: first intervention, with the orange arrow indicating the location of the PAVM. Right panel: second intervention, showing placement of embolization coils (blue arrow). PAVM: Pulmonary arteriovenous malformation

After the procedures, she continued to experience hypoxia. An echocardiogram with bubble study showed an intrapulmonary shunt, and she underwent a third intervention. A large PAVM was noted in the inferolateral segment of the right middle lobe, with a smaller branch supplying the arteriovenous malformation from the lateral segment (Figure 4).

**Figure 4:**
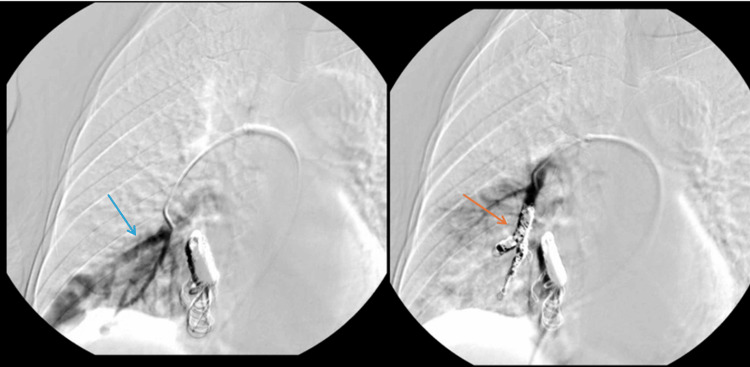
Third intervention Fluoroscopy demonstrating a large inferolateral PAVM. Left panel: Pre-second embolization, with the PAVM indicated by the blue arrow; Right panel: Post-embolization, with the occluded lesion indicated by the orange arrow. PAVM: Pulmonary arteriovenous malformation

The patient was admitted to the hospital for 14 days; no complications were reported during her hospital stay, and she was discharged home without the need for supplemental oxygen. Three weeks after discharge, the patient was evaluated in the pulmonary clinic, where she reported marked improvement in symptoms, and spirometry was obtained, which ruled out obstructive airway disease.

After two years of follow-up, the patient remained asymptomatic, and repeated CTA of the chest did not reveal new lesions.

## Discussion

PAVMs are rare abnormalities in which there is a direct connection between veins and arteries, providing communication between the pulmonary and systemic circulations that bypasses the pulmonary capillaries, producing a low-resistance, high-flow, continuous right-to-left shunt that impairs gas exchange [1,2].

PAVMs are most commonly present in patients with HHT. Acquired causes like case trauma, cardiothoracic surgery, metastatic cancer, liver cirrhosis, mitral stenosis, and infection, account for approximately 20% of the cases. Idiopathic cases represent the minority [2].

PAVMs can be classified as simple or complex based on the pulmonary artery involved. When a single segmental artery supplies the PAVMs, it is referred to as a simple PAVM; when two or more segmental pulmonary arteries supply the PAVMs, it is referred to as a complex PAVM [2-4]. According to some studies, the majority of patients with non-HHT present with a single PAVM (89%), while the presence of two or more PAVMs can be present in around 22% of the cases [5-7].

This entity presentation varies from asymptomatic to experiencing decreased exercise tolerance, persistent dyspnea, chronic hypoxia, and cyanosis, the latter being reported in less than two percent of the cases [3,5]. Massive hemoptysis and hemothorax can also occur. Other complications include paradoxical embolization of bacteria and thrombi, leading to transient ischemic attack (TIA), stroke, and brain abscess [5-7].

CTA is the preferred diagnostic modality, as it can identify, localize, and characterize the lesion, which is important for planning therapeutic management. It has been reported that contrast transthoracic echocardiography (TTE) has a sensitivity of 98.6%; however, it is mostly used as a screening tool to identify the shunt and assess risks [4].

The treatment of choice is embolization; its effectiveness has been proven since the 1980s, with a high success rate and low complication rate [8]. Invasive surgery interventions like local excision, lobectomy, and pneumonectomy are also performed, but these are reserved for those cases that are unsuitable for embolization [9,10]. Untreated cases of PAVM can have mortality as high as 50% [11].

## Conclusions

The diagnosis of PAVM can resemble a complex puzzle. It might be tempting to attribute its signs and symptoms to a simpler, more common process; however, this can lead to missed or delayed diagnosis. We cannot let the simplicity of a common diagnosis overshadow the need for a thorough and systematic evaluation. 

In this case, we identified PAVM as the cause of our patient's respiratory symptoms; thus, the patient received appropriate treatment, achieving symptom resolution with no recurrence in two years of follow-up.
